# Associations between Ambient Fine Particulate Levels and Disease Activity in Patients with Systemic Lupus Erythematosus (SLE)

**DOI:** 10.1289/ehp.1002123

**Published:** 2010-09-22

**Authors:** Sasha Bernatsky, Michel Fournier, Christian A. Pineau, Ann E. Clarke, Evelyne Vinet, Audrey Smargiassi

**Affiliations:** 1 Division of Clinical Epidemiology and; 2 Division of Rheumatology, McGill University Health Centre, Montreal, Quebec, Canada; 3 Direction de santé publique de l’Agence de la santé et des services sociaux de Montréal, Montreal, Quebec, Canada; 4 Division of Clinical Immunology and Allergy, McGill University Health Centre, Montreal, Quebec, Canada; 5 Département de santé environnementale et de santé au travail, Université de Montréal, Montreal, Quebec, Canada; 6 Institut national de santé publique du Québec, Montréal, Quebec, Canada

**Keywords:** air pollution, antibodies, disease activity, PM_2.5_, SLE, SLEDAI-2K, systemic lupus erythematous

## Abstract

**Background:**

Systemic lupus erythematosus (SLE) is a chronic disease of unclear etiology, characterized by an overactive immune system and the production of antibodies that may target normal tissues of many organ systems, including the kidneys. It can arise at any age and occurs mainly in women.

**Objective:**

Our aim was to evaluate the potential influence of particulate matter (PM) air pollution on clinical aspects of SLE.

**Methods:**

We studied a clinic cohort of SLE patients living on the island of Montreal, followed annually with a structured clinical assessment. We assessed the association between ambient levels of fine PM [median aerodynamic diameter ≤ 2.5 μm (PM_2.5_)] measured at fixed-site monitoring stations and SLE disease activity measured with the SLE Disease Activity Index, version 2000 (SLEDAI-2K), which includes anti–double-stranded DNA (anti-dsDNA) serum-specific autoantibodies and renal tubule cellular casts in urine, which reflects serious renal inflammation. We used mixed effects regression models that we adjusted for daily ambient temperatures and ozone levels.

**Results:**

We assessed 237 patients (223 women) who together had 1,083 clinic visits from 2000 through 2007 (mean age at time of first visit, 41.2 years). PM_2.5_ levels were associated with anti-dsDNA and cellular casts. The crude and adjusted odds ratios (reflecting a 10-μg/m^3^ increase in PM_2.5_ averaged over the 48 hr prior to clinical assessment) were 1.26 [95% confidence interval (CI), 0.96–1.65] and 1.34 (95% CI, 1.02–1.77) for anti-dsDNA antibodies and 1.43 (95% CI, 1.05–1.95) and 1.28 (0.92–1.80) for cellular casts. The total SLEDAI-2K scores were not associated with PM_2.5_ levels.

**Conclusions:**

We provide novel data that suggest that short-term variations in air pollution may influence disease activity in established autoimmune rheumatic disease in humans. Our results add weight to concerns that pollution may be an important trigger of inflammation and autoimmunity.

Systemic lupus erythematosus (SLE) is a chronic autoimmune disease of unclear etiology, with a prevalence as high as 1 in 2,500 women (Bernatsky et al. 2007). It is characterized by an overactive immune system that targets normal tissue in nearly any body organ. The resulting inflammation causes dysfunction and damage; involvement of major organs such as the kidneys can be particularly devastating and even life-threatening (Bernatsky et al. 2007).

The factors driving SLE are complex, but it is clear that there are definite, albeit ill-defined, exogenous triggers. Different possible environmental triggers have been considered, but at present there are relatively few relevant studies (Parks and Cooper 2006) and essentially no work has examined the effect of air pollution on SLE manifestations. This is an important knowledge gap, because there is a growing interest in pollution emissions and particulate matter (PM) exposures and their effects on health. This interest stems from recent data that have suggested that these exposures may be important triggers of systemic inflammation that could have important effects in terms of autoimmunity. Recent data have suggested that these exposures may be important triggers of systemic inflammation [for a review, see U.S. Environmental Protection Agency (EPA) 2004] that could have important effects in terms of autoimmunity.

Our aim in this study was to evaluate the potential influence of PM air pollution on the clinical course of SLE. We have focused on the effects of variations in levels of fine ambient PM with median aerodynamic diameter ≤ 2.5 μm (PM_2.5_). The main sources of PM_2.5_ in the region of the study include road-vehicle emissions and industrial emissions. Such small particles enter the body through airways and can affect immune system pathways (U.S. EPA 2004).

## Materials and Methods

### Subjects and overview of health assessments

We studied subjects who were followed in a clinical registry at the Lupus Clinic of the McGill University Health Centre (MUHC). All patients in this registry cohort fulfilled the revised American College of Rheumatology (ACR) criteria for SLE (Hochberg 1997; Tan et al. 1982). Subjects in the cohort completed an annual evaluation that consisted of a review of symptoms, medications, physical findings, and laboratory testing. The data were used to construct validated measures of disease activity [SLE Disease Activity Index, version 2000 (SLEDAI-2K)] (Gladman et al. 2002) and damage [Systemic Lupus International Collaborating Clinics/ACR Damage Index (SLICC/ACR)] (Gladman et al. 2002). The SLEDAI-2K is a “weighted” index that provides a measurement of disease activity of the organ systems in SLE over a 10-day period before the annual evaluation. The index includes central nervous system features, vascular involvement, kidney disease, musculoskeletal disease, dermatological features, serosal involvement, immune system activity, hematological features, and constitutional symptoms (see Appendix). Theoretically, patients can score a maximum of 105, but in practice, scores greater than 45 are unusual. In the present study, we analyzed data collected on patients who resided on the island of Montreal (Quebec, Canada) and who were evaluated from January 2000 through September 2007. All subjects consented to be included in the registry, and studies were conducted under ethical approval from the MUHC.

We studied associations between PM_2.5_ and the SLEDAI-2K total score. We were also specifically interested in the presence or absence of renal tubule cellular casts, which are a marker for severe kidney inflammation related to SLE, and the presence or absence of antibodies against double-stranded DNA (anti-dsDNA). Anti-dsDNA antibodies are specific for SLE, although they are not present in all patients, and correlate with various types of system involvement (musculoskeletal, hematological, immunological, and, most important, renal disease) (Mosca et al. 2006). In the clinical setting, serial measurements of anti-dsDNA antibodies are routinely performed to identify and predict SLE activity and disease flare-ups, although the utility of this approach in decision making and treatment remains under discussion (Tseng et al. 2006).

### Disease activity measurements

The physical examinations of the SLE patients were performed by a specialist physician who (along with the patient) was blinded to the potential associations (pollution and disease activity) being tested. Hematological and immunological manifestations of SLE (including the presence or absence of anti-dsDNA antibodies) were evaluated using venous peripheral blood samples collected between 0830 and 1130 hours on the day of the assessment. Urinalyses (to assess kidney disease, including microscopic observation of casts) were done on freshly voided specimens. The presence of anti-dsDNA was assessed, up to 2007, using a radioimmunoassay method based on the Farr technique (Manthorpe et al. 1978). Thereafter, an enzyme-linked immunosorbent assay technique was used (Farrzyme human high-avidity anti-dsDNA enzyme immunoassay kit; Binding Site, Birmingham, UK).

We studied the two specific laboratory measures (anti-dsDNA antibodies and renal casts, both of which contribute to the SLEDAI-2K total score) because they are well known as important markers of the most active and severe forms of SLE and may represent more objective elements of SLE activity (as opposed to the musculoskeletal manifestation of arthritis for example, which may be a subtle or even subjective finding). Furthermore, these laboratory elements were measured specifically on the day of the clinical evaluation, in contrast with other components of the SLEDAI-2K (e.g., dermatological manifestations and oral ulcers) that are scored positive if a patient reports that they were present at any time within the 10 days before the clinicial evaluation, even if they are absent at the time of the annual examination.

### Measures of ambient PM_2.5_ and ozone levels

Hourly measurements at fixed-site monitoring stations on the island of Montreal were obtained from the Environment Canada’s National Air Pollution Surveillance network (http://www.etc-cte.ec.gc.ca/napsdata). The networks use tapered-element oscillating microbalance and beta attenuation monitor samplers (Ville de Montréal 2010) for PM_2.5_ measurements and ultraviolet absorption analyzers for ozone. Hourly PM_2.5_ concentrations were averaged across all Montreal stations; these mean hourly levels, averaged for the 24 hr preceding each clinical evaluation (from 1000 hours the day before the clinical evaluation to 0900 hours the day of the evaluation) and for up to 10 days before, were assigned to each patient. Ozone levels on the days before the evaluation dates were averaged over the 8 hr from 0900 to 1700 hours because ozone levels are highest during these hours. The median numbers of monitoring stations used to compute the daily PM_2.5_ and ozone concentrations were 7 and 10, respectively.

### Outdoor ambient temperatures

We computed the mean of the outdoor temperatures for the 24 hr preceding each clinic evaluation and for prior days (from 1000 hours the day before the clinical evaluation to 0900 hours the day of the evaluation). Hourly temperature data were acquired from the Environment Canada Meteorological Centre located at the Pierre Elliott Trudeau International Airport (Dorval, Quebec, Canada), about 20 km from the city core (Environment Canada 2010).

### Analysis

To analyze associations between PM_2.5_ exposure levels and SLEDAI-2K total score, anti-dsDNA antibodies, and urinary casts, we used random intercept models for repeated-measures (longitudinal) data to account for correlations among repeated measures within individuals. Thus, the interpretation of reported estimates is at the subject level. We used negative binomial mixed models for SLEDAI-2K total score and logistic mixed models to estimate associations with binary outcome variables (anti-dsDNA and renal casts). The SLEDAI-2K score is a discrete and an asymmetrical variable. As overdispersion was present in the distribution of this variable (likelihood ratio test, *p* < 0.001) that precluded the use of a Poisson model, so we used a negative binomial model. Measures of association [incidence rate ratios (IRRs) for SLEDAI-2K scores and odds ratios (ORs) for anti-dsDNA and renal casts] and their 95% confidence intervals (CIs) are presented as associations per 10-μg/m^3^ increases in PM_2.5_ levels. Adjusted models included ozone levels [a potential risk factor for inflammation (e.g., Khatri et al. 2009) that may vary with PM_2.5_] and ambient temperatures (to account for seasonal effects) averaged over the same time window as PM_2.5_.

Components of the SLEDAI-2K could be scored positive if present at any time within the 10 days preceding the assessment. Thus, to assess exposure–response relationships for total SLEDAI-2K scores, we assessed effects not only for PM_2.5_ levels on the day before the evaluation (average of hourly levels measured from 1000 hours the day before to 0900 hours on the day of the visit), but also for hourly averages of up to 10 days before the visit.

Age at SLE onset, age at time of physician assessment, smoking (ever vs. never), race/ethnicity (with separate dummy variables for Caucasian, black, or Asian), education (whether or not the subject had any secondary schooling vs. ≤ 11 years), and daily use of immunomodulatory drugs (hydroxychloroquine, mycophenolate, oral or parenteral methotrexate, azathioprine, and prednisone) were evaluated as potential effect modifiers by adding product terms between each factor and PM_2.5_ to models. The effects of interactions were tested with α = 0.05. These variables did not vary with PM_2.5_ levels (data not shown) and so were not considered confounders. We performed all analyses with STATA (version 10.1; StataCorp LP, College Station, TX, USA).

## Results

We studied 237 patients (223 women, 14 men) with a mean age at the time of their first MUHC visit of 41.2 years old (range, 18–83 years; Table 1). During the study period (January 2000–September 2007), each patient participated in an average of 4.6 assessments that averaged 409 days apart. For this period, the PM_2.5_ levels rarely exceeded the PM_2.5_ Canadian objective of 30 μg/m^3^ (Table 2).

**Figure 1:**
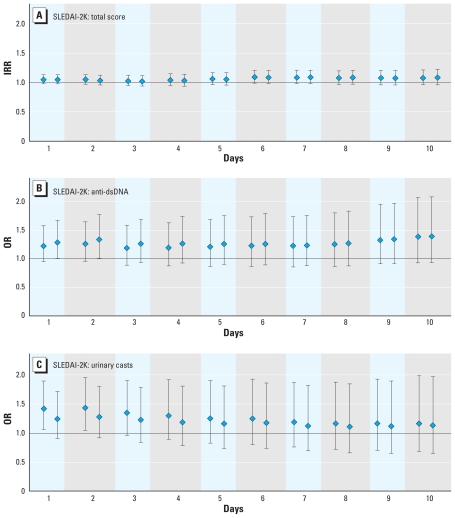
Associations between PM_2.5_ levels averaged over the day before the medical evaluations (from 1000 hours the day before the evaluation to 0900 hours on the day of the evaluation) and up to the 10 days before, and SLEDAI-2K scores: total score (*A*), anti-dsDNA (*B*), and urinary casts (*C*). Associations are expressed per 10 μg/m^3^ of PM_2.5_. In each pair of data points, the first represents crude estimates and the second coefficients adjusted for ozone and temperature levels at the same exposure window as PM_2.5_. Whiskers represent 95% CIs.

The intraclass correlation for the three outcome variables within individual patients ranged from 0.12 to 0.40. IRR estimates did not clearly demonstrate a relationship between a 10-μg/m^3^ increase in PM_2.5_ and total SLEDAI-2K scores (Figure 1A), although our results suggested some potential effect with PM_2.5_ levels averaged over 10 days (not evident for PM_2.5_ levels on the day before the evaluation; crude IRR for PM_2.5_ levels averaged over 6 days = 1.09; 95% CI, 0.99–1.20). Anti-dsDNA and urinary casts were significantly associated with PM_2.5_ levels shortly before the clinical visits (averaged over 24 or 48 hr before; Figure 1B,C). The crude OR relative to an increase in PM_2.5_ of 10 μg/m^3^ (48-hr averages) was 1.26 (95% CI, 0.96–1.65) for the presence of anti-dsDNA and 1.43 (95% CI, 1.05–1.95) for the presence of renal casts. Although not statistically significant, there was also a suggestion of some association between anti-dsDNA and PM_2.5_ levels averaged over 10 days. Controlling for ambient temperatures and ozone levels had little influence (association with PM_2.5_ levels averaged over 48 hr: anti-dsDNA, OR = 1.34; 95% CI, 1.02–1.77; renal casts, OR = 1.28, 0.92–1.80). Including potential effect modifiers in the model (e.g., medications and race/ethnicity) and examinations for interactions failed to clearly demonstrate important or consistent effects at α = 0.05.

## Discussion

To our knowledge, the results presented here are the first to suggest that autoimmune inflammatory diseases such as SLE may be associated with variations in air pollutant levels. These findings add to a multitude of studies that have consistently related the adverse health effects (both acute and chronic) of ambient fine PM (Brook 2008; Calderón-Garcidueñas et al. 2008; Ghio et al. 2000; U.S. EPA 2004). This study also adds weight to concerns that ambient air pollutants may be an important trigger of inflammation and autoimmunity.

Fine particles are believed to induce effects through inflammation and oxidative stress in the lungs when inhaled (U.S. EPA 2004). The smaller particles have also been suggested to enter the systemic circulation (Nemmar et al. 2002), where they would be capable of causing widespread inflammation and oxidative stress (Shukla et al. 2000; U.S. EPA 2004). The proinflammatory circulating cytokines generated upon air pollution exposure (Shoenfelt et al. 2009; van Eeden et al. 2001; Watterson et al. 2007) could plausibly act to trigger and/or heighten autoimmune disease.

Interestingly, there are two recent studies that have suggested road traffic and pollution as triggers of autoimmune rheumatic diseases in humans, such as juvenile idiopathic arthritis (JIA) and rheumatoid arthritis (RA). In one study, Zeft et al. (2009) reported that increased concentrations of PM_2.5_ and stagnant air conditions were associated with significantly elevated risk of the onset of JIA in young children. In another study, Hart et al. (2009) found that the risk of onset of RA for participants in the Nurses’ Health Study was higher for women who lived within 50 m of a major road than for those who lived 200 m or farther away; this finding suggests that pollution from traffic is indeed an environmental risk factor for autoimmune diseases such as RA.

We observed associations between short-term variations in PM_2.5_ levels (averaged over 1–10 days) and anti-dsDNA and urinary casts that suggest acute effects. Although it is physiologically plausible for urinary casts and anti-dsDNA to vary on a daily basis with PM_2.5_ levels, studies are needed to document the kinetics of these features and to support the observed associations. Such knowledge could help the understanding of their diagnostic value. We did not clearly demonstrate an association between PM_2.5_ and overall SLEDAI-2K total scores; in part this may be due to the way the SLEDAI-2K is scored and to the fact that some features of disease activity could have been present early in the 10-day window preceding the assessment (but not actually present at the time of the visit). Because the associations we observed were mainly with SLEDAI-2K lab measures (i.e., urinary casts and anti-dsDNA), further studies are needed to address whether fine PM levels are mainly associated with renal lupus activity or with the general disease activity.

There are several limitations associated with how we estimated exposure of our patients to PM_2.5_. We used PM_2.5_ daily regional averages because we did not have access to personal exposure information. We assumed that all subjects were in Montreal for the 10 days before their visits, but we did not have information on their actual location. Furthermore, outdoor ambient PM_2.5_ levels may not adequately represent exposure of our patients because people are likely to spend most of their time indoors, where both indoor and outdoor PM_2.5_ sources contribute to indoor levels (World Health Organization 2000). Future studies on the association between short-term variations in air pollution and inflammatory disease activity should address these limitations.

Because we did not study incident disease and because the patients under study were all from one tertiary care center, we cannot comment on potential roles of long-term exposure or spatial variations in air pollution. Nor did we assess potential effects of pollution on the actual incidence of SLE. However, future work by our team, using population-based administrative data, will explore these issues.

## Conclusion

Our data suggest that short-term variations in air pollution may influence disease activity in established autoimmune rheumatic disease in humans. Our results add weight to concerns that pollution may be an important trigger of inflammation and autoimmunity. Although further studies are needed to confirm these findings, we suspect that clinicians, health authorities, and patients may need to examine and address the potentially serious adverse health effects of pollution.

## Figures and Tables

**Table 1:** Characteristics of lupus patients (*n* = 237).

Characteristic	Value
Age (years)	
Disease onset	31.0 ± 13.8
First clinical visit	41.2 ± 15.5
Women	223 (94.1)
Race/ethnicity	
Caucasians	150 (63.3)
Blacks	37 (15.6)
Asians	30 (12.7)
Other	20 (8.8)
Ever-smoker^a^	101 (44.1)
Education ≤ high school^b^	55 (27.2)
No. of assessments per patient	4.6 ± 2.5

Data are reported as mean ± SD or *n* (%).

a Eight subjects were missing smoking status.

b Thirty-five subjects were missing education level.

**Table 2:** SLEDAI-2K scores, medication use, and environmental measurements on days of patients’ clinical evaluations (*n* = 1,083 visits).

Characteristic	Value
Medication	
Hydroxychloroquine	729 (67.3)
Mycophenolate	119 (11.0)
Methotrexate (oral)	41 (3.8)
Methotrexate (parenteral)	11 (1.0)
Azathioprine	73 (6.7)
Prednisone	174 (16.1)
Disease activity	
Total SLEDAI-2K score (*n* = 1,003)	4.5 ± 4.8 (0–28)
Anti-dsDNA, patient evaluations with positive assay (*n* = 957)	263 (27.5)
Renal tubule casts present on urinalysis (*n* = 861)	81 (9.4)
Disease damage SLICC/ACR score (*n* = 1,005)	1.9 ± 2.2
Environmental variables	
PM_2.5_ (μg/m^3^)	10.0 ± 7.8 (1.1–54.9)
Ozone (μg/m^3^)	47.7 ± 23.7 (2.3–137.7)
Temperature (°C)	7.5 ± 11.4 (−21.5 to −28.8)

Data are reported as *n* (%) or mean ± SD (range).

**Appendix 1:** SLEDAI-2K data collection sheet. (Check weight in SLEDAI-2K score column if descriptor is present at the time of the visit or in the preceding 10 days.)

Weight (check)	Descriptor	Definition
8 ❑	Seizure	Recent onset, exclude metabolic, infectious, or drug causes.
8 ❑	Psychosis	Altered ability to function in normal activity due to severe disturbance in the perception of reality. Include hallucinations, incoherence, marked loose associations, impoverished thought content, marked illogical thinking, bizarre, disorganized, or catatonic behavior. Exclude uremia and drug causes.
8 ❑	Organic brain syndrome	Altered mental function with impaired orientation, memory, or other intellectual function, with rapid onset and fluctuating clinical features, inability to sustain attention to environment, plus at least two of the following: perceptual disturbance, incoherent speech, insomnia or daytime drowsiness, or increased or decreased psychomotor activity. Exclude metabolic, infectious, or drug causes.
8 ❑	Visual disturbance	Retinal changes of SLE. Include cytoid bodies, retinal hemorrhages, serous exudates or hemorrhages in the choroids, or optic neuritis. Exclude hypertension, infection, or drug causes.
8 ❑	Cranial nerve disorder	New onset of sensory or motor neuropathy involving cranial nerves.
8 ❑	Lupus headache	Severe, persistent headache; may be migrainous but must be nonresponsive to narcotic analgesia.
8 ❑	Cerebrovascular accident	New onset of cerebrovascular accident(s); exclude arteriosclerosis.
8 ❑	Vasculitis	Ulceration, gangrene, tender finger nodules, periungual infarction, splinter hemorrhages, or biopsy or angiogram proof of vasculitis.
4 ❑	Arthritis	Two or more joints with pain and signs of inflammation (i.e., tenderness, swelling, or effusion).
4 ❑	Myositis	Proximal muscle aching/weakness, associated with elevated creatine phosphokinase/aldolase or electromyogram changes or a biopsy showing myositis.
4 ❑	Urinary casts	Heme granular or red blood cell casts.
4 ❑	Hematuria	More than five red blood cells/high power field; exclude stone, infection, or other cause.
4 ❑	Proteinuria	> 0.5 g/24 hr.
4 ❑	Pyuria	More than five white blood cells/high power field; exclude infection.
2 ❑	Rash	Inflammatory type rash.
2 ❑	Alopecia	Abnormal, patchy, or diffuse loss of hair.
2 ❑	Mucosal ulcers	Oral or nasal ulcerations.
2 ❑	Pleurisy	Pleuritic chest pain with pleural rub or effusion or pleural thickening.
2 ❑	Pericarditis	Pericardial pain with at least one of the following: rub, effusion, or electrocardiogram or echocardiogram confirmation.
2 ❑	Low complement	Decrease in the complement proteins C3 and C4 or in total complement activity (CH50), below the lower limit of normal for testing laboratory.
2 ❑	Increased DNA binding	Increased DNA binding by Farr assay above normal range for testing laboratory.
1 ❑	Fever	> 38°C; exclude infectious cause.
1 ❑	Thrombocytopenia	< 100,000 platelets/×10^9^/L; exclude drug causes.
1 ❑	Leukopenia	< 3,000 white blood cells/×10^9^/L; exclude drug causes.

	Total score	

Reproduced with permission from Gladman et al. (2002).
